# Separation of the main flavonoids and essential oil from seabuckthorn leaves by ultrasonic/microwave-assisted simultaneous distillation extraction

**DOI:** 10.1098/rsos.180133

**Published:** 2018-07-18

**Authors:** Chunying Li, Jingjing Zhang, Chunjian Zhao, Lei Yang, Wenyan Zhao, Hongwei Jiang, Xueting Ren, Weiran Su, Yuzheng Li, Jiajing Guan

**Affiliations:** 1 Key Laboratory of Forest Plant Ecology, Ministry of Education, Northeast Forestry University, Harbin 150040, People's Republic of China; 2 State Engineering Laboratory of Bio-Resource Eco-Utilization, Northeast Forestry University, Harbin 150040, People's Republic of China

**Keywords:** seabuckthorn, flavonoids, ultrasound/microwave-assisted extraction, ionic liquid, essential oil

## Abstract

Volatile essential oils (EOs), non-volatile rutin (RU), quercetin (QU), kaempferol (KA) and isorhamnetin (IS) were effectively extracted and isolated from seabuckthorn (*Hippophae rhamnoides* L.) leaves by ionic liquid-based ultrasound/microwave-assisted simultaneous distillation extraction (ILUMASDE). After optimization by response surface methodology, EOs, RU, QU, KA and IS were separated under the following optimum conditions: an ionic liquid of 1.0 M 1-butyl-3-methyl imidazole bromine salt ([C4mim]), liquid/solid ratio of 12 ml g^−1^, extraction time of 34 min, microwave power of 540 W and a fixed ultrasonic power of 50 W. Under the optimized conditions of ILUMASDE, the extraction yields of RU, QU, KA, IS and EOs were 9.18 ± 0.35, 5.52 ± 0.23, 3.03 ± 0.11, 5.64 ± 0.24 mg g^−1^ and 0.095 ± 0.004%, respectively. The yield of EOs obtained using ILUMASDE was 1.07-fold higher than that obtained by conventional hydrodistillation extraction (HDE). In addition, the components of the EOs obtained using ILUMASDE and HDE were similar. The extraction yields of RU, QU, KA, IS obtained by ILUMASDE were 1.03–1.35-fold higher than that obtained by the ethanol ultrasonic-assisted extraction (EUAE), ionic liquid-based ultrasonic-assisted extraction (ILUAE) and ionic liquid-based microwave-assisted extraction (ILMAE). And the extraction time used by ILUMASDE was 34 min, which is 14.17%, 56.67%, 56.67% and 85.00% less than those used by HDE, EUAE, ILUAE and ILMAE, respectively. Therefore, ILUMASDE can be considered a rapid and efficient method for extracting flavonoids and EO from seabuckthorn (*Hippophae rhamnoids* L.) leaves.

## 1. Introduction

Seabuckthorn (*Hippophae rhamnoides* L.), a deciduous shrub or dwarf tree of the Euphorbiaceae, is distributed in the temperate zone, cold temne and subtropical high mountain area of Eurasian continent [1,2], and is rich in a variety of bioactive substances, including flavonoids, triterpenoids, sterols, lipids and volatile oil, etc. [3–5]. These active substances have strong protecting cardio-cerebrovascular, enhancing immune, lowering blood lipid, anti-oxidation, anti-ageing, anti-tumour and anti-inflammatory properties [6–8]. Seabuckthorn is widely used in health food and pharmaceutical industry [9,10].

**Figure 1.**
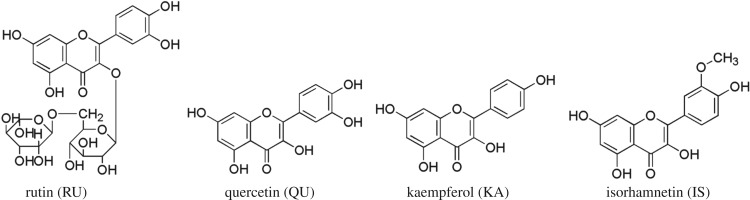
Structures of four flavonoids in the extract of seabuckthorn leaves.

The flavonoids in seabuckthorn are mainly rutin (RU), isorhamnetin (IS), quercetin (QU) and kaempferol (KA), etc., which are present in many parts of seabuckthorn, and there are the highest amounts of them in foliage [11]. Seabuckthorn flavonoids have anti-ageing, anti-fatigue, anti-tumour, enhancing immunity bioactivity [12,13]. The molecular structures of RU, QU, KA and IS are shown in figure 1.

Volatile oils, also known as essential oils (EOs), are very important natural components occurring in seabuckthorn, which has the cell regenerating, anti-inflammatory and photo-protective activity with promising applications in dermatology and cosmetics [4,14,15].

Although some studies on EOs in seabuckthorn berries have been found, there have been few reports on volatile components in leaves [16–18].

There are some reports on extracting volatile EOs and non-volatile phenolics in separate procedures. The most common extraction methods for flavonoids and phenolic acids are soaking extraction [19], hot water extraction [20], ultrasound-assisted extraction (UAE) [21–23], microwave-assisted extraction (MAE) [24–26] and enzyme-assisted extraction [27]. EOs have been extracted using various conventional methods, such as organic solvent extraction [28], steam distillation extraction (SDE) [29], supercritical fluid extraction [30], microwave-assisted extraction [31] and hydrodistillation extraction (HDE) [32]. However, the general application of traditional techniques above has been limited by several shortcomings, including time-consuming, poor recoveries, degradation of target compounds, atmospheric pollution [33,34]. To overcome those shortcomings, an efficient extraction method is imperative to be considered in this study.

Ionic liquids (ILs) as a new type of green solvent to replace traditional volatile organic solvents have lots of advantages, such as low melting point, high thermal stabilities, better solubility, low vapour pressure, controllable polarity, low toxicity and recyclability [35,36].

Nowadays, simultaneously ultrasonic/microwave-assisted extraction (UMAE), which combined the advantage of both ultrasonic and microwave, is a prevailing separation method and widely applied in food and pharmaceutical industry [37,38].

Conventional SDE combines steam distillation with continuous extraction using a solvent or a mixture of solvents, which is considered to be superior to other methods for obtaining EOs. The microwave-assisted distillation-solvent extraction of EO has also been developed [39]. Unfortunately, this technique could only be used for liquid–liquid extraction of volatile compounds, and could not be applied to non-volatile ingredients such as flavonoids.

With respect to the advantages of ILs, UMAE and SDE, and on the basis of the previous work carried out by our research team, an ionic liquid-based ultrasonic/microwave-assisted simultaneous distillation and extraction (ILUMASDE) method for separating of the main flavonoids and EO from seabuckthorn leaves was proposed [40,41]. Response surface methodology (RSM) with a Box–Behnken design (BBD) was used to evaluate and optimize the conditions that could influence the yields of target components, including the concentration of IL, liquid/solid ratio, extraction time and microwave power. Additionally, the yields of EOs, RU, QU, KA and IS obtained with the proposed technique were compared with conventional extraction methods.

## 2. Experimental set-up

### 2.1. Reagents and materials

ILs ([C_2_mim]Br, [C_4_mim]Br, [C_6_mim]Br, [C_8_mim]Br, [C_10_mim]Br, [C_4_mim][BF_4_], [C_4_mim][HSO_4_], [C_4_mim][NO_3_], [C_4_mim]Cl, [C_4_mim][ClO_4_]; C_2_mim=1-ethyl-3-methylimidazolium, C_4_mim=1-butyl-3-methylimidazolium, C_6_mim=1-hexyl-3-methylimidazolium, C_8_mim=1-octyl-3-methylimidazolium, C_10_mim=1-decyl-3-methylimidazolium) were purchased from the Chengjie Chemical Co., Ltd (Shanghai, China). CA, RU, QU, KA and IS standards were purchased from Sigma company (USA).

Formic acid, methanol and acetonitrile of HPLC grade were purchased from J&K Chemical Ltd (Beijing, China). Deionized water was acquired by a Milli-Q system (Millipore, Billerica, MA, USA). All solutions prepared for HPLC analysis were filtered through a 0.45 µm nylon membrane (Guangfu Chemical Reagents Co.,Tianjin, China) and degassed by ultrasonication in advance. Seabuckthorn (*Hippophae rhamnoides* L.) leaves were obtained from Heilongjiang Academy of Agricultural Sciences (Heilongjiang, China). They were dried in the shade at room temperature, comminuted using a disintegrator (HX-200A, Yongkang Hardware and Medical Instrument Plant, Yongkang, China), passed through a stainless-steel sieve (80 mesh), and stored in closed desiccators at room temperature before use.

### 2.2. ILUMASDE

**Figure 2.**
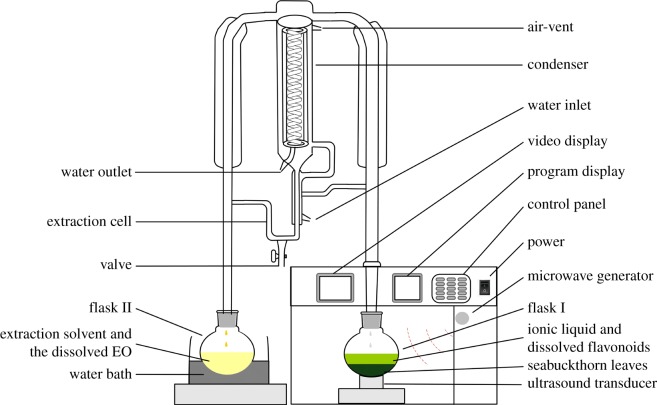
Schematic of the ILUMASDE device.

In the ILUMASDE device (figure 2) the Likens-Nickerson SDE unit (Chaoyue Laboratory Instrument Works Co., Shanghai, China) is connected to the UMAE system (CW-2000, Shanghai Xintuo Analytical, China Shanghai Instrument Technology Co., Ltd). First, the seabuckthorn leaves powder 20.0 g were passed through an 80 mesh sieve and then added to the reaction flask (I) together with the ionic liquid, mixed by a stirring bar, and 50 ml of dichloromethane was put in a 100 ml flask (II). Flask I was subjected to ultrasonic/microwave treatment in the oven of the ILUMASDE apparatus and flask II was heated in a water bath at 45°C. After the extraction, the mixture in flask I was rapidly cooled to room temperature using a cold bath, filtered through a filter paper and then the filtrate was filtered through a 0.45 µm microporous membrane for subsequent HPLC analysis. The organic solvent in flask II was collected and dried using anhydrous sodium sulfate. EOs were obtained by removing solvent under reduced pressure, then were sealed and stored at 4°C for GC-MS analysis.

### 2.3. Conventional extraction procedure

#### 2.3.1. HDE for EOs

In total, 20.0 g of seabuckthorn leaves powders and 200 ml of water were placed in the reaction flask, then the reaction flask was connected to the hydrodistillation unit. The reactor was heated for 4 h using an electric heating set, EOs were separated from the water, dried with anhydrous sodium sulfate, and then sealed at 4°C. Three operation replicates were performed.

#### 2.3.2. Ethanol ultrasonic-assisted extraction (EUAE) for the main flavonoids

In total, 20.0 g seabuckthorn leaves powder were mixed with 200 ml of 60% (V:V) ethanol and then RU, QU, KA and IS were extracted by UAE method. The extracts were filtered through a filter paper after 60 min of extraction time. The filtrate was filtered through a 0.45 µm microporous membrane for subsequent HPLC analysis. Three operation replicates were performed.

#### 2.3.3. Ionic liquid-based ultrasonic-assisted extraction (ILUAE) for the main flavonoids

In total, 20.0 g seabuckthorn leaves powder and appropriate ionic liquid solution 200 ml were mixed, and then UAE method was used to extract RU, QU, KA and IS with 200 W of ultrasonic power, 60°C extraction temperature, 60 min of extraction time. The filtrate was filtered through a 0.45 µm microporous membrane for subsequent HPLC analysis. Three operation replicates were performed.

#### 2.3.4. Ionic liquid-based microwave-assisted extraction (ILMAE) for the main flavonoids

Seabuckthorn leaves powder 20.0 g and appropriate ionic liquid solution 200 ml were mixed, RU, QU, KA and IS were extracted by MAE method with 500 W of microwave power, 10 ml g^−1^ of liquid/solid ratio and 40 min of extraction time. The filtrate was filtered through a 0.45 µm microporous membrane for subsequent of HPLC analysis. Three operation replicates were performed.

### 2.4. Experimental design

#### 2.4.1. ILUMASDE optimization using RSM

In order to obtain the optimal extraction effect of ILUMASDE, the main parameters of the extraction experiment were optimized under the condition of the optimum ionic liquid. Three main influencing factors were selected from the single-factor experiments as independent variables, i.e. liquid/solid ratio (X_1_), reaction time (X_2_) and microwave power (X_3_). The effects of three factors on the extraction yields of four flavonoids and volatile oil in seabuckthorn leaves were studied using a three-factor-three-level BBD followed by RSM analysis. The second-order multivariate regression equation of the extraction yields of four flavonoids and EO in seabuckthorn leaves is as follows:

2.1 $$Y=\beta_{0}+\sum_{i=1}^{3}\beta_{i}X_{i}+\sum_{i=1}^{3}\beta_{ii}X_{i}^{2}+\sum_{i=1}^{3}\sum_{j=i+1}^{3}\beta_{ij}X_{i}X_{j},$$

Where *X_i_* and *X_j_* represent independent variables, *Y* is the response variable, *β*_0_, *β_i_*_,_ *β_ii_* and *β_ij_* are the constants, regression coefficients of one term, quadratic terms and interaction terms, respectively. The actual and coded levels of the independent variables used in the experimental design are shown in table 1. All the experiments were repeated three times and the yields were given as average values.

**Table 1.** Experimental conditions used in the BBD analysis and the corresponding measured responses.

	coded			actual		
run	factor A (*X*_1_)	factor B (*X*_2_)	factor C (*X*_3_)	liquid/solid ratio, ml g^−1^ (*X*_1_)	time, min (*X*_2_)	microwave power, W (*X*_3_)
1	1	0	1	14	30	700
2	0	0	0	10	30	500
3	1	−1	0	14	10	500
4	−1	0	1	6	30	700
5	0	1	−1	10	50	300
6	0	0	0	10	30	500
7	0	−1	1	10	10	700
8	0	1	1	10	50	700
9	0	0	0	10	30	500
10	0	0	0	10	30	500
11	0	−1	−1	10	10	300
12	1	0	−1	14	30	300
13	−1	−1	0	6	10	500
14	1	1	0	14	50	500
15	−1	1	0	6	50	500
16	−1	0	−1	6	30	300
17	0	0	0	10	30	500

#### 2.4.2. Statistical analysis

Statistical analysis for the experimental design and data were carried out using Design-Expert (Version 7.0, Stat-Ease Inc., Minneapolis, MN) software. The fit quality of the polynomial model equation was expressed by the regression coefficient (*R*^2^) and the lack of fit, the Fisher test value (*F*-value) and their interactions were estimated using analysis of variance. The adequacy of the fitted model was evaluated by comparing the actual and predicted values. The optimum values for three variables were obtained by statistical analysis (DE software, 8.01 version, Stat-Ease, Inc., Minneapolis, MN, USA).

### 2.5. GC-MS analysis of EOs

The EOs was analysed by a gas chromatography–mass spectrometry system (Agilent 7890A-Agilent 7000B, Agilent, Santa Clara, CA) with HP-5MS column (30 m × 0.25 mm × 0.25 µm). The GC conditions were: high purity helium (≥99.9%) at 1.0 ml min^−1^ as carrier gas, the split injection ratio 20 : 1, the injection volume 1 µl, the injection temperature of 20°C and the oven temperature was increased from 70°C to 200°C at 2°C min^−1^, then increased to 240°C at a rate of 8°C min^−1^ and finally to 280°C at a speed of 2°C min^−1^, then kept 30 min there. The MS conditions were: electron energy 70 eV, emission current 100 mA, ion source (EI) temperature 200°C, mass scanning range 50–350 *m/z*, solvent delay 4 min. The results were analysed by NIST Library and searching and retention index (RI) value. The EOs components were identified by comparing their RIs and mass spectral fragmentation patterns with those of compounds in the National Institute of Standards and Technology (NIST) mass spectral library, and with MS data reported in the literature [42].

### 2.6. HPLC determination of RU, QU, KA and IS

**Figure 3.**
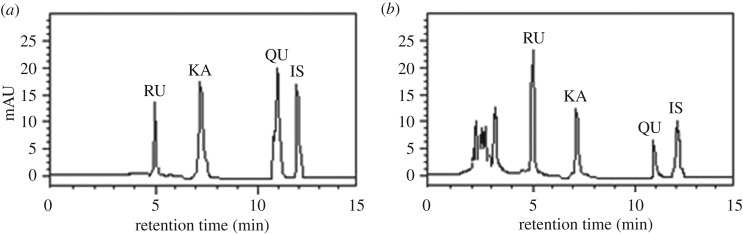
HPLC chromatograms of standard for RU, QU, KA and IS (*a*) and extraction sample (*b*).

The chromatographic system (Jasco, Tokyo, Japan) consisted of a HiQ Sil-C18 reversed-phase column (4.6 mm × 250 mm, 5 µm), a PU 980 pump and 1575 UV detector. The HPLC conditions were as follows: the mobile phase was methanol-acetonitrile-water (40 : 15 : 45, v/v/v) containing 1% formic acid, the column temperature was maintained at 30°C, the detection wavelength was 368 nm, the flow rate was 1.0 ml min^−1^, the injection volume was 10 µl, and the run time was 15 min. HPLC chromatograms of standards and sample are shown in figure 3.

### 2.7. Scanning electron microscopy observation

The effect of the different extraction method on the microstructure of the plant material was observed using scanning electron microscopy (SEM). The dried extraction samples obtained after treatments by different methods were observed using a scanning electron microscope (Quanta-200 SEM, FEI Co., Hillsboro, OR, USA). The samples were fixed on aluminium stubs using adhesive tape then sputtered with gold using a sputter coater. All the samples were scanned under high vacuum conditions at an accelerating voltage of 12.5 kV (500× magnification).

## 3. Results and discussion

### 3.1. Choosing an appropriate IL

**Figure 4.**
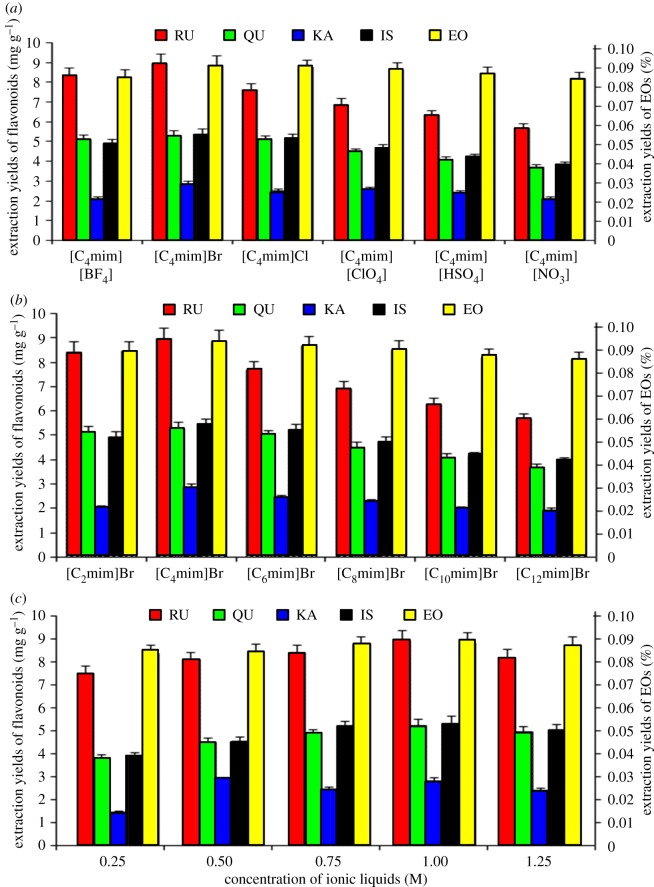
Effects of IL anions (*a*) and cations (*b*) and concentration of [C_4_mim]Br (*c*) on yields of RU, QU, KA, IS and EOs.

The anion is considered to be the significant factor influencing the properties of ILs [35]. 1-Alkyl-3-methylimidazolium-based ILs are widely used in sample preparation and compounds extraction from plant materials, thus, 1-Butyl-3-methylimidazolium-based ILs with the same concentrations but six different anions (Br^−^, Cl^−^, BF4^−^, ClO_4_^−^, HSO_4_^−^ and NO_3_^−^) were used in UMASDE to identify the best IL anion [43]. The results (figure 4*a*) show that the yields of both EOs and the four flavonoids obtained using [C_4_mim]Br was higher than those obtained using other ILs. The results reflect the higher ability of the [C_4_mim]Br to dissolve cellulose in plant cells, which involves having the inter- and intra-molecular hydrogen bonds dissociated and new hydrogen bonds between carbohydrate hydroxyl protons and the IL anions formed [44,45]. Br^−^ was therefore chosen as the IL anion for extracting RU, QU, KA, IS and EOs from seabuckthorn leaves.

The length of IL alkyl chain affects water miscibility, and thus affects the extraction efficiency of compounds [46]. 1-Alkyl-3-methylimidazolium ILs containing the same anion, i.e. Br^−^, but different lengths (ethyl to decyl) of alkyl chains in the cation, were used in UMASDE to evaluate the effect of the alkyl chain length. The results (figure 4*b*) show that the cation alkyl chain length significantly affects the yields of the target compounds and the highest yields of all the five target compounds were obtained using [C_4_mim]Br. This may be due to the difference of nano-structuring of alkyl domains within the IL, which affected the intermolecular forces between IL and the target compounds. Based on the above analysis, [C_4_mim]Br was considered to be the best IL for use in further experiments.

This may be due to the hydrogen bonds formed by [C_4_mim]Br and the target compounds, which affected van der Waals force between them.

### 3.2. Optimization of IL concentration

The effect of [C_4_mim]Br solution concentration (from 0.25 to 1.25 M) on the yields of target compounds was studied. The results are shown in figure 4*c*. It can be clearly seen that the yields of RU, QU, KA, IS and EOs increased significantly with increasing concentration from 0.25 to 1 M. This phenomenon may be due to the increasing destruction extent of cell wall along with growing concentration of [C_4_mim]Br. However, with further increases in the [C_4_mim]Br concentration, i.e. from 1 to 1.25 M, slight decreases in the yields of targets can be observed. The reason that a larger concentration of [C_4_mim]Br leads to a decrease in the extraction yield may be because the higher concentration of the IL increases the viscosity of the solvent and the diffusion of the solvent. Above all, 1 M [C_4_mim]Br was identified as the most appropriate solvent for the use in subsequent experiments.

### 3.3. Parameter optimization by RSM

#### 3.3.1. Model building and statistical analysis

To optimize the interactions between the three variables (liquid/solid ratio X_1_, reaction time X_2_ and microwave power X_3_) and the yields of RU, QU, KA, IS and EOs, 17 experiments were performed. The experimental design and the results are shown in tables 1 and 2. The results for each dependent variable and their coefficients of determination (*R*^2^) indicated that the proposed models were adequate, there was no significant lack of fit, and the *R*^2^ for the yields were satisfactory (table 3). A model is well fitted to the experimental data when the model *F*-value is significant but the lack of fit is non-significant [47]. The model *F*-values were 67.41, 160.70, 93.43, 52.19 and 124.16 for RU, QU, KA, IS and EOs, respectively, indicating that the models were significant. In addition, Lack of fit *F*-tests gave values for RU, QU, KA, IS and EOs of 2.13, 1.39, 2.34, 2.72 and 2.15, respectively. These values implied that the lack of fit was not significant. The probabilities for the occurrence of the lack of fit *F*-values were 0.24%, 0.37%, 0.21%, 0.18% and 0.24% for RU, QU, KA, IS and EOs, respectively. These values could be treated as statistical noise, indicating excellent agreement of the experimental values with the predicted values. It was deduced that the models were appropriate to predict the responses based on the factors mentioned above. A second-order polynomial model was used to express the yields of RU, QU, KA, IS and EOs using the following equations:

3.1 $$\begin{array}{rl} Y_{1} & \;=\text{13}\text{.47}+\text{0}\text{.98}X_{1}+\text{1}\text{.46}X_{2}+\text{1}\text{.17}X_{3}-\text{0}\text{.23}X_{1}X_{2}+\text{0}\text{.41}X_{1}X_{3}\\ & \;\;-\text{0}\text{.28}X_{2}X_{3}-\text{1}\text{.41}X_{1}^{2}-\text{1}\text{.22}X_{2}^{2}-\text{1}\text{.16}X_{3}^{2}, \end{array}$$

3.2 $$\begin{array}{rl} Y_{2} & \;=0.48+6.58\times 10^{-2}X_{1}+6.52\times 10^{-2}X_{2}+8.43\times 10^{-2}X_{3}+1.79\\ & \;\;\times 10^{-2}X_{1}X_{2}+2.51\times 10^{-3}X_{1}X_{3}-3.78\times 10^{-3}X_{2}X_{3}-6.11\\ & \;\;\times 10^{-2}X_{1}^{2}-8.72\times 10^{-2}X_{2}^{2}-6.04\times 10^{-2}X_{3}^{2}, \end{array}$$

3.3 $$\begin{array}{rl} Y_{3} & \;=1.93+0.17X_{1}+0.23X_{2}+0.19X_{3}+0.13X_{1}X_{2}+9.81\times 10^{-2}X_{1}X_{3}\\ & \;\;+5.23\times 10^{-2}X_{2}X_{3}-0.25X_{1}^{2}-0.21X_{2}^{2}-0.15X_{3}^{2}, \end{array}$$

3.4 $$\begin{array}{rl} Y_{4} & \;=0.36+4.04\times 10^{-2}X_{1}+3.77\times 10^{-2}X_{2}+4.62\times 10^{-2}X_{3}+2.24\\ & \;\;\times 10^{-2}X_{1}X_{2}+2.53\times 10^{-2}X_{1}X_{3}-3.81\times 10^{-3}X_{2}X_{3}-6.30\\ & \;\;\times 10^{-2}X_{1}^{2}-5.32\times 10^{-2}X_{2}^{2}-3.79\times 10^{-2}X_{3}^{2} \end{array}$$

3.5 $$\begin{array}{rl} \text{and}\;Y_{5} & \;=3.51\times 10^{-2}+3.32\times 10^{-3}X_{1}+4.77\times 10^{-3}X_{2}+4.82\times 10^{-3}X_{3}-3.50\\ & \;\;\times 10^{-4}X_{1}X_{2}+3.83\times 10^{-4}X_{1}X_{3}+5.01\times 10^{-4}X_{2}X_{3}\\ & \;\;-4.12\times 10^{-3}X_{1}^{2}-5.53\times 10^{-3}X_{2}^{2}-6.80\times 10^{-3}X_{3}^{2}, \end{array}$$

where *Y*_1_, *Y*_2_, *Y*_3_, *Y*_4_ and *Y*_5_ are yields of RU, QU, KA, IS and EOs, respectively. *X*_1_ is the liquid/solid ratio (ml g^−1^), *X*_2_ is the extraction time (min) and *X*_3_ is the microwave power (W).

**Table 2.** Experimental and predicted results for the yields of RU, QU, KA, IS and EOs.

	experimental value					predicted value				
no.	RU (mg g^−1^)	QU (mg g^−1^)	KA (mg g^−1^)	IS (mg g^−1^)	EOs (%)	RU (mg g^−1^)	QU (mg g^−1^)	KA (mg g^−1^)	IS (mg g^−1^)	EOs (%)
1	8.57	5.37	2.77	5.34	0.084	8.75	5.43	2.82	5.33	0.085
2	8.93	5.05	2.68	5.20	0.091	8.76	5.09	2.75	5.35	0.091
3	7.03	3.30	1.82	2.99	0.062	6.88	3.35	1.82	3.15	0.063
4	7.05	3.89	2.02	3.51	0.065	6.94	3.98	2.06	3.70	0.065
5	7.56	3.26	2.17	3.77	0.055	7.58	3.37	2.23	3.93	0.057
6	8.94	5.04	2.71	5.25	0.092	8.76	5.09	2.75	5.35	0.091
7	7.23	3.89	2.16	4.31	0.060	7.21	3.78	2.11	4.16	0.058
8	8.78	5.10	2.94	5.22	0.085	8.74	5.07	2.90	5.19	0.085
9	8.61	5.24	2.81	5.47	0.092	8.76	5.09	2.75	5.35	0.091
10	8.66	5.15	2.77	5.28	0.093	8.76	5.09	2.75	5.35	0.091
11	5.27	1.89	1.69	2.65	0.036	5.32	1.93	1.73	2.67	0.035
12	6.58	3.68	2.06	3.41	0.058	6.70	3.60	2.01	3.22	0.057
13	5.17	2.31	1.72	2.96	0.042	5.31	2.34	1.73	2.93	0.044
14	8.62	5.12	2.85	4.93	0.088	8.48	5.10	2.85	4.96	0.086
15	7.36	3.37	2.00	3.56	0.072	7.51	3.32	2.00	3.40	0.071
16	6.14	2.31	1.86	3.07	0.043	5.95	2.25	1.81	3.07	0.042
17	8.64	4.94	2.75	5.50	0.088	8.76	5.09	2.75	5.35	0.091

**Table 3.** The variance analysis of response surface model for extraction yield of RU, QU, KA, IS and EOs.

source	sum of squares	degree of freedom	mean square	*F*-value	*p*-value	remarks
*RU*						
model	59.56	9	6.62	67.41	<0.0001	significant
*X*_1_	7.64	1	7.64	77.87	<0.0001	significant
*X*_2_	17.08	1	17.08	174.01	<0.0001	significant
*X*_3_	10.97	1	10.97	111.79	<0.0001	significant
*X*_1_*X*_2_	0.21	1	0.21	2.16	0.1855	not significant
*X*_1_*X*_3_	0.67	1	0.67	6.85	0.0346	significant
*X*_2_*X*_3_	0.32	1	0.32	3.25	0.1143	not significant
$X_{1}^{2}$	8.39	1	8.39	85.42	<0.0001	significant
$X_{2}^{2}$	6.25	1	6.25	63.71	<0.0001	significant
$X_{3}^{2}$	5.65	1	5.65	57.59	<0.0001	significant
residual	0.69	7	9.84 × 10^−2^			
lack of fit	0.42	3	0.14	2.13	0.2396	not significant
pure error	0.26	4	6.58 × 10^−2^			
Cor total	60.25	16				
CV = 2.68%		*R*^2^ = 0.9886		Adj *R*^2^ = 0.9739		predicted *R*^2^ = 0.8810
*QU*						
model	0.20	9	2.22 × 10^−2^	160.70	<0.0001	significant
*X*_1_	3.52 × 10^−2^	1	3.52 × 10^−2^	258.76	<0.0001	significant
*X*_2_	3.38 × 10^−2^	1	3.38 × 10^−2^	246.25	<0.0001	significant
*X*_3_	5.73 × 10^−2^	1	5.73 × 10^−2^	418.14	<0.0001	significant
*X*_1_*X*_2_	1.33 × 10^−3^	1	1.33 × 10^−3^	9.78	0.0167	significant
*X*_1_*X*_3_	2.50 × 10^−5^	1	2.50 × 10^−5^	0.18	0.6812	not significant
*X*_2_*X*_3_	5.63 × 10^−5^	1	5.63 × 10^−5^	0.41	0.5409	not significant
$X_{1}^{2}$	1.62 × 10^−2^	1	1.62 × 10^−2^	114.84	<0.0001	significant
$X_{2}^{2}$	3.17 × 10^−2^	1	3.17 × 10^−2^	235.05	<0.0001	significant
$X_{3}^{2}$	1.50 × 10^−2^	1	1.50 × 10^−2^	111.10	<0.0001	significant
residual	9.54 × 10^−4^	7	1.36 × 10^−4^			
lack of fit	4.86 × 10^−4^	3	1.62 × 10^−4^	1.39	0.3681	not significant
pure error	4.67 × 10^−4^	4	1.17 × 10^−4^			
Cor total	0.20	16				
CV = 3.03%		*R*^2^ = 0.9952		Adj *R*^2^ = 0.9890		predicted *R*^2^ = 0.9570
*KA*						
model	1.63	9	0.18	93.43	<0.0001	significant
*X*_1_	0.22	1	0.22	115.78	<0.0001	significant
*X*_2_	0.41	1	0.41	213.59	<0.0001	significant
*X*_3_	0.27	1	0.27	141.24	<0.0001	significant
*X*_1_*X*_2_	7.02 × 10^−2^	1	7.02 × 10^−2^	36.23	0.0005	significant
*X*_1_*X*_3_	3.83 × 10^−2^	1	3.83 × 10^−2^	19.61	0.0030	significant
*X*_2_*X*_3_	1.14 × 10^−2^	1	1.14 × 10^−2^	5.69	0.0485	not significant
$X_{1}^{2}$	0.26	1	0.26	136.56	<0.0001	significant
$X_{2}^{2}$	0.18	1	0.18	91.95	<0.0001	significant
$X_{3}^{2}$	9.59 × 10^−2^	1	9.59 × 10^−2^	49.36	0.0002	significant
residual	1.36 × 10^−2^	7	1.94 × 10^−3^			
lack of fit	8.65 × 10^−3^	3	2.88 × 10^−3^	2.34	0.2143	not significant
pure error	4.92 × 10^−3^	4	1.23 × 10^−3^			
Cor total	1.64	16				
CV = 2.67%		*R*^2^ = 0.9917		Adj *R*^2^ = 0.9811		Predicted *R*^2^ = 0.9111
*IS*						
model	7.77 × 10^−2^	9	8.64 × 10^−3^	52.19	<0.0001	significant
*X*_1_	7.04 × 10^−3^	1	7.04 × 10^−3^	42.53	0.0003	significant
*X*_2_	1.23 × 10^−2^	1	1.23 × 10^−2^	69.80	<0.0001	significant
*X*_3_	1.71 × 10^−2^	1	1.71 × 10^−2^	100.46	<0.0001	significant
*X*_1_*X*_2_	1.98 × 10^−3^	1	1.98 × 10^−3^	11.97	0.0106	significant
*X*_1_*X*_3_	2.42 × 10^−3^	1	2.42 × 10^−3^	14.60	0.0065	significant
*X*_2_*X*_3_	5.63 × 10^−5^	1	5.63 × 10^−5^	0.34	0.5782	not significant
$X_{1}^{2}$	1.70 × 10^−2^	1	1.70 × 10^−2^	100.46	<0.0001	significant
$X_{2}^{2}$	1.18 × 10^−2^	1	1.18 × 10^−2^	70.16	<0.0001	significant
$X_{3}^{2}$	6.03 × 10^−3^	1	6.03 × 10^−3^	36.42	0.0005	significant
residual	1.16 × 10^−3^	7	1.66 × 10^−4^			
lack of fit	7.77 × 10^−4^	3	2.59 × 10^−4^	2.72	0.1792	not significant
pure error	3.81 × 10^−4^	5	9.53 × 10^−5^			
Cor total	7.90 × 10^−2^	16				
CV = 4.54%		*R*^2^ = 0.9853		Adj *R*^2^ = 0.9664		predicted *R*^2^ = 0.8348
*EOs*						
Model	8.98 × 10^−4^	9	9.94 × 10^−5^	124.16	<0.0001	significant
*X*_1_	8.91 × 10^−5^	1	8.91 × 10^−5^	111.28	<0.0001	significant
*X*_2_	1.82 × 10^−4^	1	1.82 × 10^−4^	227.78	<0.0001	significant
*X*_3_	1.87 × 10^−4^	1	1.87 × 10^−4^	233.78	<0.0001	significant
*X*_1_*X*_2_	4.90 × 10^−7^	1	4.90 × 10^−7^	0.61	0.4597	not significant
*X*_1_*X*_3_	5.63 × 10^−7^	1	5.63 × 10^−7^	0.70	0.4297	not significant
*X*_2_*X*_3_	1.00 × 10^−6^	1	1.00 × 10^−6^	1.25	0.3007	not significant
$X_{1}^{2}$	6.98 × 10^−5^	1	6.98 × 10^−5^	87.21	<0.0001	significant
$X_{2}^{2}$	1.25 × 10^−4^	1	1.25 × 10^−4^	156.03	<0.0001	significant
$X_{3}^{2}$	1.96 × 10^−4^	1	1.96 × 10^−4^	244.74	<0.0001	significant
residual	5.61 × 10^−6^	7	8.01 × 10^−7^			
lack of fit	3.46 × 10^−6^	3	1.15 × 10^−6^	2.15	0.2371	not significant
pure error	2.15 × 10^−6^	4	5.37 × 10^−7^			
Cor total	9.01 × 10^−4^	16				
CV = 3.32%		*R*^2^ = 0.9938		Adj *R*^2^ = 0.9858		predicted *R*^2^ = 0.9348

#### 3.3.2. Analysis of the response contour

**Figure 5.**
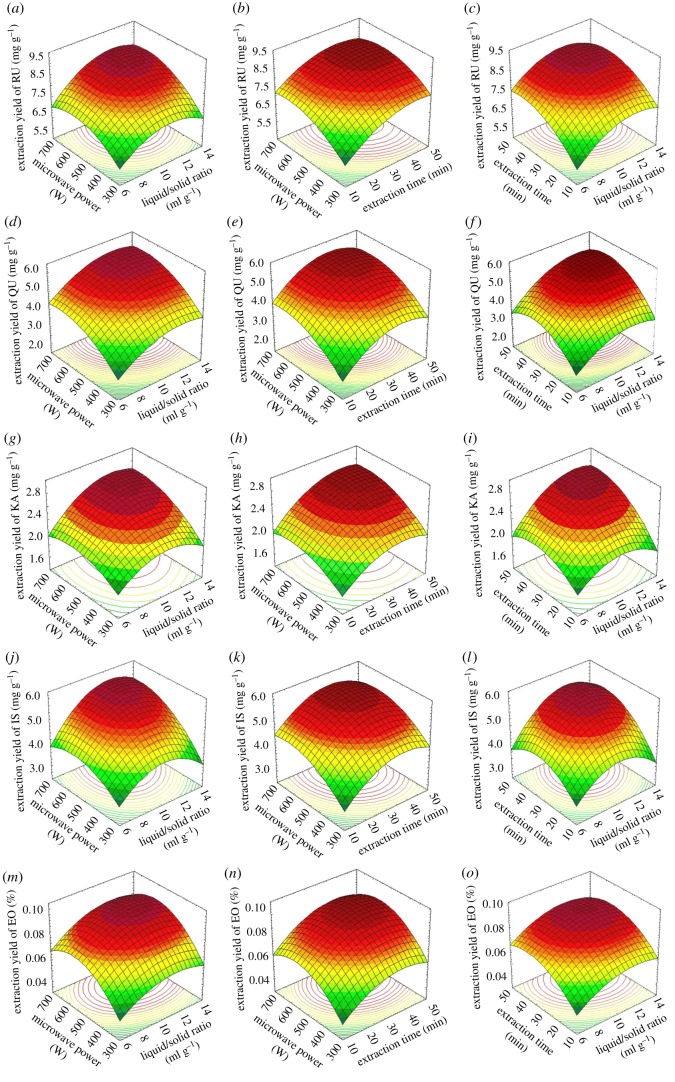
Response surface plots showing the effects of variables (liquid/solid ratio, extraction time and microwave power) on the extraction yields of RU (*a–c*), QU (*d–f*), KA (*g–i*), IS (*j–l*) and EOs (*m–o*).

The effects of the independent variables and their interactions on the yields of RU, QU, KA, IS and EOs are shown in figure 5. It can be seen from figure 5 that the yield of target compounds increased first and then decreased with the increase of the liquid/solid ratio, microwave power and extraction time.

With the increase of liquid/solid ratio, the concentration difference of the target compounds in the liquid and solid phase increased, thus improving the mass transfer of materials to solvents, yields of the target compounds increased.

However, the liquid/solid ratio increases further, which decreases the rate of temperature rise. At lower temperature, it is unfavourable to dissolve and diffuse the target component in the liquid phase. Thereby, the yield of target compounds increased first and then decreased with the increase of the liquid/solid ratio.

From figure 5, different microwave power resulted in the different yields of target compounds. This increase in the yields was attributed to the fact that microwave irradiation energy can enhance the extent to which the solvent penetrates into the solid matrix of the powdered sample [48]. However, too high microwave power caused an excessively high temperature inside the plant material, which destroyed the composition of the target compounds and led to the loss of volatile oil in the experimental process. Therefore, the yield of target compounds increased first and then decreased gradually with the microwave power.

Extraction time is a crucial parameter in solvent extraction for natural active ingredients. From figure 5, the yield of target compounds increased first and then decreased with the increase of extraction time, which is mainly due to the fact that, within a certain period of time, the longer extraction time made the target compounds into solvents completely. But the longer time led to decomposition of the target compounds, thus the extraction yield of target compounds decreased [49].

The optimum conditions of RU, QU, KA, IS and EOs for the maximum predicted yields by software were, respectively: liquid/solid ratio of 10.5 ml g^−1^ for RU, 10.8 ml g^−1^ for QU, 13.4 ml g^−1^ for KA, 11.2 ml g^−1^ for IS and 11.7 ml g^−1^ for EOs; extraction time of 32.7 min for RU, 34.0 min for QU, 32.7 min for KA, 37.5 min for IS; 9.09 mg g^−1^ for RU, 5.42 mg g^−1^ for QU, 3.14 mg g^−1^ for KA, 5.72 mg g^−1^ for IS and 0.095% for EOs.

### 3.4. Verification test under optimum conditions

The optimum extraction conditions (independent variables) obtained using the DE software were as follows: extraction solvent 1 M [C_4_mim]Br, extraction time 34 min, liquid/solid ratio 12 ml g^−1^ and microwave power 540 W. The suitability of the model equations for predicting the response values was confirmed by performing a verification experiment under these optimized conditions. The actual yields of RU, QU, KA, IS and EOs were 9.18 ± 0.35 mg g^−1^, 5.52 ± 0.23 mg g^−1^, 3.03 ± 0.11 mg g^−1^, 5.64 ± 0.24 mg g^−1^ and 0.095 ± 0.004%, respectively. The RU, QU, KA, IS and EOs yields obtained using ILUMASDE were close to the predicted values and showed low deviations (less than 1.2%), demonstrating the reliability of the RSM models.

### 3.5. Comparison of different extraction methods

#### 3.5.1. Effect of different extraction method on the yield of RU, QU, KA and IS

**Figure 6.**
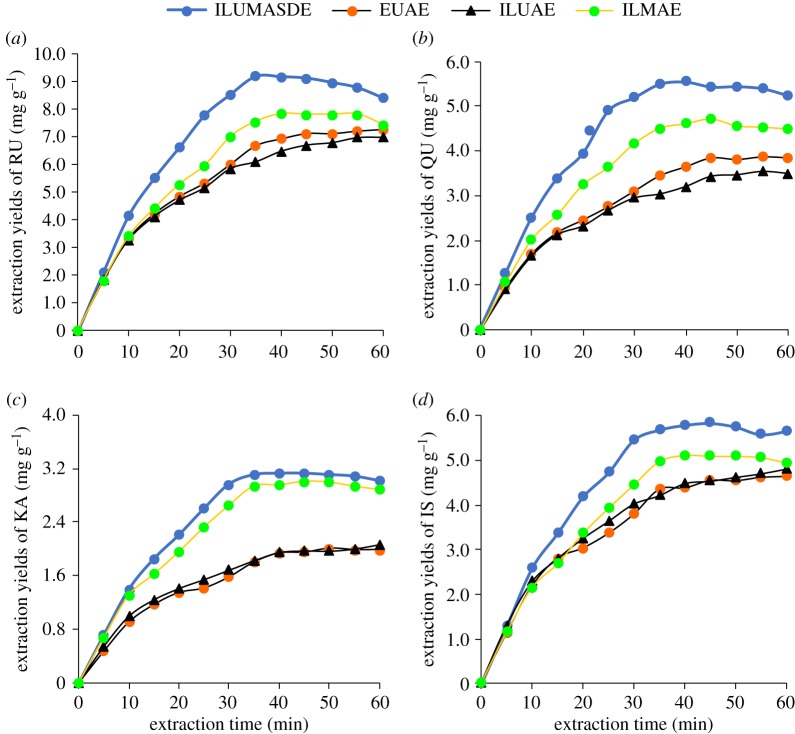
Comparison of four different method for extraction of flavonoid.

A comparison of ILUMASDE with other methods (ethanol ultrasonic-assisted extraction (EUAE), ionic liquid-based ultrasonic-assisted extraction (ILUAE) and ionic liquid-based microwave-assisted extraction (ILMAE)) was performed based on the yields of RU, QU, KA and IS (figure 6). The yield of the four flavonoids obtained by ILUMASDE was much higher than those obtained by the other methods. It may be the IL dissolves the cellulose in the plant cell walls and the target compounds are highly soluble in the IL. In addition, the ultrasonic/microwave combination breaks the plant cells, which also accelerates the release of RU, QU, KA and IS from the matrix and it also increases the molecular motion of the extraction solvent, which increases mass transfer, leading to higher yields of the target compounds.

#### 3.5.2. Effect of different extraction method on yields and components of EOs

**Figure 7.**
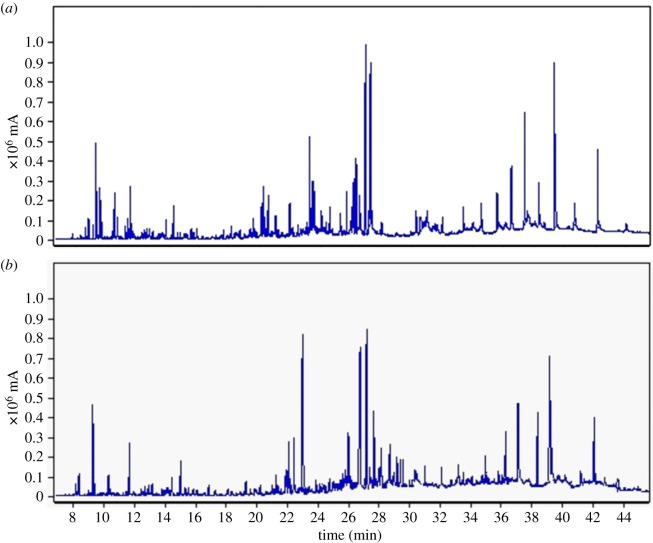
GC-MS chromatogram of EOs in seabuckthorn leaves by ILUMASDE (*a*) and HDE (*b*).

Experimental results show that the EOs yields were 0.095 ± 0.004% by ILUMASDE and 0.089 ± 0.003% by HDE, respectively. The yield of EOs obtained by ILUMASDE was much higher than that obtained by HDE method. The relative contents of individual volatile components are expressed as percentages for their peak areas relative to the total peak area. The results of GC-MS analysis showed that the volatile components in the EOs of ILUMASDE were almost the same as that obtained by HDE, and 24 of the volatile components were detected in the EOs by ILUMASDE (figure 7*a*) and HDE (figure 7*b*). The major components of EOs obtained by the two methods were myristic acid, (Z)-8-dodecen-1-yl acetate, methyl palmitelaidate, α-linolenic acid, linolenic acid, stearic acid and palmitic acid (table 4). Therefore, ILUMASDE can effectively extract EOs from the seabuckthorn leaves without changing the components.

**Table 4.** Chemical compositions of EOs obtained by ILUMASDE.

				relative peak area (%)	
no.	retention time (min)	compounds	RI	ILUMASDE	HDE
1	9.253	lauryl alcohol	1449	5.64 ± 0.12	6.25 ± 0.12
2	11.517	benzaldehyde	1497	2.98 ± 0.07	3.52 ± 0.12
3	13.854	myristyl alcohol	1546	1.72 ± 0.05	1.68 ± 0.03
4	14.335	lauric acid	1556	2.68 ± 0.07	2.43 ± 0.05
5	20.235	celery alcohol	1664	2.67 ± 0.06	2.56 ± 0.06
6	20.545	tetradecanol	1670	2.68 ± 0.05	2.92 ± 0.06
7	21.965	heptadecene	1694	1.75 ± 0.03	1.66 ± 0.02
8	23.199	tridecanoicacid	1714	1.12 ± 0.02	1.39 ± 0.02
9	23.483	3-[3,3-pentylacetate]-cyclohexanone	1719	6.02 ± 0.09	6.52 ± 0.10
10	23.570	tetramethylhexadecanol	1720	1.75 ± 0.01	1.86 ± 0.02
11	24.626	eicosane	1738	1.35 ± 0.01	1.32 ± 0.02
12	25.692	tert-butyl benzenedicarboxylate	1756	2.89 ± 0.05	2.78 ± 0.04
13	26.093	octadecane	1763	2.02 ± 0.04	2.55 ± 0.03
14	26.927	myristic acid	1776	10.24 ± 0.15	8.33 ± 0.17
15	30.978	cetylaldehyde	1846	2.82 ± 0.01	2.98 ± 0.02
16	32.162	pentadecanoic acid	1868	1.45 ± 0.02	1.82 ± 0.03
17	35.582	methyl hexadecanoate	1925	3.24 ± 0.02	3.28 ± 0.03
18	36.508	triadane	1942	3.74 ± 0.03	3.96 ± 0.04
19	37.375	methyl palmitelaidate	1956	6.98 ± 0.05	5.79 ± 0.04
20	38.314	palmitic acid	1973	4.82 ± 0.06	3.93 ± 0.07
21	39.392	(Z)-8-dodecen-1-yl acetate	1991	9.47 ± 0.08	9.22 ± 0.09
22	42.195	Α-linolenic acid	2143	5.55 ± 0.05	4.37 ± 0.04
23	43.237	linolenic acid	2154	6.46 ± 0.06	4.87 ± 0.05
24	43.574	stearic acid	2188	5.25 ± 0.04	4.33 ± 0.04
		total identified		95.29	90.32

#### 3.5.3. Scanning electron microscopy

**Figure 8.**
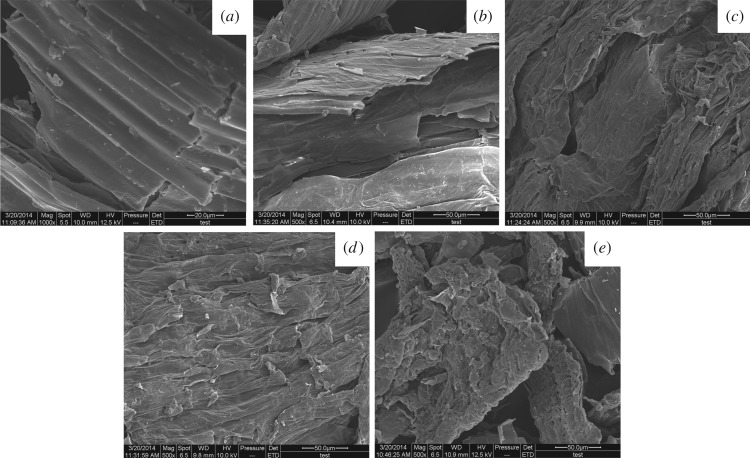
Scanning electron microscopic images of seabuckthorn leaves samples. (*a*): raw materials; (*b–e*) show samples treated by EUAE, ILUAE, ILMAE and ILUMASDE, respectively.

To investigate the correlation between extraction yield and cell wall breakage, SEM was used to observe the structure of untreated samples and those extracted using the different extraction methods (EUAE, ILUAE, ILMAE and ILUMASDE). The different method produced great physical changes on the tissue of the seabuckthorn leaves (figure 8*a–e*, respectively). Figure 8*a* clearly shows that the external surface of the untreated sample tissues was intact and smooth. After EUAE treatment, some cells were slightly damaged (figure 8*b*), more being destroyed by ILUAE (figure 8*c*) and ILMAE (figure 8*d*), but most of the cells were completely disrupted and collapsed after ILUMASDE treatment (figure 8*e*). This indicated that ILUMASDE ruptured cell walls more effectively, thus resulting in a higher extraction yield of the flavonoids and EOs in seabuckthorn leaves.

## 4. Conclusion

ILUMASDE was proposed to isolate and quantify EOs and non-volatile RU, QU, KA and IS from the leaves of seabuckthorn. Satisfactory yields for the five target components were obtained through optimization by RSM with a BBD. Compared with traditional methods, ILUMASDE gives higher yields of target components while consuming a shorter extraction time. And it is worth mentioning that ILUMASDE accelerates the isolation of EOs without causing major changes in the EOs composition. Volatile and non-volatile active compounds co-exist in many plants. Simultaneous extraction of non-volatile and volatile compounds from plants can reduce the operation steps of the extraction process. Therefore, the proposed effective ILUMASDE method is a promising technique for the simultaneous extraction of non-volatile and volatile compounds from other plants.

## Data accessibility

This article has no additional data.
